# Designing the next generation of medicines for malaria control and eradication

**DOI:** 10.1186/1475-2875-12-187

**Published:** 2013-06-06

**Authors:** Jeremy N Burrows, Rob Hooft van Huijsduijnen, Jörg J Möhrle, Claude Oeuvray, Timothy NC Wells

**Affiliations:** 1 Medicines for Malaria Venture (MMV), PO Box 1826, route de Pré-Bois 20, Geneva 15 1215, Switzerland

**Keywords:** Malaria, Plasmodium, Anopheles, Drug discovery, Medicines, Target candidate profile, Target product profile, MMV

## Abstract

In the fight against malaria new medicines are an essential weapon. For the parts of the world where the current gold standard artemisinin combination therapies are active, significant improvements can still be made: for example combination medicines which allow for single dose regimens, cheaper, safer and more effective medicines, or improved stability under field conditions. For those parts of the world where the existing combinations show less than optimal activity, the priority is to have activity against emerging resistant strains, and other criteria take a secondary role. For new medicines to be optimal in malaria control they must also be able to reduce transmission and prevent relapse of dormant forms: additional constraints on a combination medicine. In the absence of a highly effective vaccine, new medicines are also needed to protect patient populations. In this paper, an outline definition of the ideal and minimally acceptable characteristics of the types of clinical candidate molecule which are needed (target candidate profiles) is suggested. In addition, the optimal and minimally acceptable characteristics of combination medicines are outlined (target product profiles). MMV presents now a suggested framework for combining the new candidates to produce the new medicines. Sustained investment over the next decade in discovery and development of new molecules is essential to enable the long-term delivery of the medicines needed to combat malaria.

## The need for target product profiles in malaria drug discovery and development

Malaria is a critical public health challenge, historically being responsible for the deaths of millions, particularly young children and expectant mothers. The past decade has seen significant progress in the control of malaria, with a reduction in reported cases [1]. There were 655,000 deaths reported in 2010 from over 200 million cases, down from over a million a decade ago. This success has been accomplished mostly by the expanded use of combination medicines, insecticide-treated nets (ITNs) and indoor residual spraying (IRS). Provided that current levels of political and financial support for malaria control are sustained, these numbers are expected to continue to fall over the next decade with increased distribution of control measures and with the potential of a vaccine being launched in 2015 [2]. In parallel with this progress, our understanding of the biology of the parasite has entered a new era. With the sequencing of the parasite genomes [3-5], new potential drug targets have emerged. Powerful new screening and imaging technologies have also made it possible to screen millions of compounds directly against the parasite in culture. This has led to the identification of many new active molecules against the erythrocytic stages of malaria, several of which are now in clinical development, and the identification of a new generation of molecular targets [6-8].

Two major types of challenge for the development of new medicines against malaria remain: those external to the drug discovery community, and those internal. The external challenge is the changing malaria landscape. Emergence and spread of resistance are always major concerns in infectious disease, and recent reports in the literature confirm decreased patient responses to artemisinin derivatives in South-East Asia [9,10] combined with decreasing efficacy of the partner drugs used in artemisinin combination therapy (ACT) [11]. Replacements for artemisinin-based endoperoxides and combination partners are urgently required. Ideally at least one component needs to be as fast-acting as the artemisinin derivatives to provide rapid relief of symptoms (the community has come to expect this), and as affordable as chloroquine was when it was used as first-line treatment. Modeling studies underline the key role that medicines can play in malaria eradication [12,13]. Medicines can be used both to treat patients’ symptoms and cure them of acute disease, as well as prophylaxis or chemoprotection, and these can play a complementary role alongside a partially effective vaccine [12,13]. Animal studies warn that parasites which escape from a partially effective vaccine may gain in virulence in a process more sophisticated than simple antigenic drift [14]. In addition, there are indications that the mosquito vector (*Anopheles*) is developing behavioural strategies to evade ITNs [15], and resistance to the pyrethroid class of insecticides used in the nets is increasing. The cost of failure in malaria control is high: the historical experience with chloroquine and DDT resistance shows that the loss of frontline interventions can have a devastating effect on the impact of the disease if a new generation of therapies and other interventions are not available.

Other public health factors will influence the type of medicines needed in the future, such as the need for anti-malarial treatments for patients who are already receiving treatment for Human Immunodeficiency Virus (HIV), tuberculosis (TB) or other co-infections. Co-treatment for HIV infection is especially relevant due to risks for interactions between the medicines used to treat HIV and those for malaria, through interference with metabolic pathways involving cytochromes, especially P450 3A4. This increases the risks for altered pharmacokinetics leading to either reduced efficacy or enhanced drug exposure and side effects. In addition the pathology of co-infection means that such patients are especially vulnerable, and this may present additional constraints for the safety of new medicines. Finally, the chemical stability of new medicines is another key challenge. Fixed-dose artemisinin combination therapies are stable in Zone IV conditions (37°C, high relative humidity) for 2–3 years. Given the difficulties of distribution, then any improvement over this level of stability would be of considerable advantage in the future.

In addition, there are internal factors, within the community, which arise from the new goal of long-term malaria eradication. These bring additional challenges in drug discovery beyond those required by medicines that effect simple case control. First, the new medicines need to be able to reduce and, ideally, prevent transmission from one infected patient to the next (R_0_ < <1). Second, they need to safely prevent the relapses with *Plasmodium vivax* and *Plasmodium ovale*. Third, significant post-treatment prophylaxis (treatment of a malaria case providing protection against future infection) may help to reduce the clinical burden of malaria especially in high-transmission areas. Fourth, new medicines will be needed for chemoprotection (causal or chemoprophylaxis) that are necessary to protect vulnerable populations such as infants and expectant mothers. All of these medicines must be safe enough for use in sensitive patient groups, including pregnant women [12], the youngest of children [16] and patients with other co-morbidities, such as HIV and TB infection, or malnutrition, and correct doses must be selected for each groups. It is important to emphasize that due to the combination of these constraints there is unlikely to be a one-size-fits-all solution in the campaign to eliminate malaria; indeed many of these issues cannot be de-risked until Phase IV.

Typically, a potential medicine for an infectious disease should take about twelve years on the journey from the first hypothesis to registration, provided funding is not limiting. In the case of malaria, timelines for the discovery and development of new medicines are longer because of the need for combinations, but there are improvements in the early stages with some molecules moving from screening to human proof-of-concept in five years. Nonetheless, the complexity of combination therapies (which require additional steps in development), and registration in highly diverse countries means that the overall project timeline to launch in disease-endemic countries will probably remain at 12–15 years. With these long timelines it is essential to start out with a clear idea of what success will look like: what are the anticipated needs in the clinic at the time of launch, what is the ideal clinical candidate molecule, and how will these be combined, with existing therapies, into the ideal final product? This requires a clear hypothesis or hypotheses as to how the medical need will change over the next fifteen years. The description of the desired product is captured in what is known as the Target Product Profile (TPP). It is important also to underline that the target product profile can vary with different external factors. The clearest example here would be the spread of ‘artemisinin resistance’. Obviously, in countries or districts where no ACT is clinically effective, the TPP will be different from those where current therapy is still fully active. Since for malaria, the medicine, or target product will contain more than one active ingredient, the attributes of a target product profile can be divided into many different target candidate profiles (TCP), each of which is effectively the job description for a new molecule entering clinical development. Exactly how the overall list of attributes needed in the final product is divided between the different molecules it contains and indeed how many different components are needed will be a matter of much debate, and there are many different potential solutions. However, this debate will be anchored in reality when the activities of each candidate in patients is seen, a theme which is explored later in this paper. The other reality check in this process is provided by comparison to existing medications, which act as the gold standards for clinical trials in terms of safety, efficacy, potential for transmission-blocking and relapse prevention, cost of goods, and overall convenience. It is, therefore, important to ask continually which potential benefits a new molecule will bring compared with existing molecules (both those which have been approved, and those in development), throughout the discovery and lead optimization process.

After some years of stagnation, the global pipeline of malaria medicines in development is now progressing as a result of significant investments over the past decades. This fresh acceleration raises an additional complication: that the minimum requirements for new medicines are not fixed, but are continually moving. As the number of molecules with the potential to meet a particular TCP increases, then the standards for new molecules will move higher. Conversely, if clinical candidates are lost from the portfolio due to safety considerations or the emergence of resistance, the bar may be lowered, though the shortcomings of the failed drug, whether safety or efficacy, will still need to be overcome. It is likely that compounds will be parked at various stages of the preclinical and clinical development processes such that they can then be reactivated in response to factors external to each individual project, such as spread of resistance to a mainstay therapy. This underlines the need for all drug discoverers to have access to a clear and accurate picture of all molecules in development. A detailed review of the global landscape was recently published [17]. An online global malaria medicines portfolio map, updated every three months, can be found on the MMV web site [18]. Recommendations for both TPPs and TCPs need to be viewed in the context of the malERA - the Malaria Eradication Agenda, which set out to characterize the changes needed to malaria research to accommodate declining malaria incidence in some countries and the prospect of local malaria elimination. These include considerations for drug discovery [19] alongside other strategies for malaria eradication [20-28]. The strategy outlined the advantages of medicines that could be given as a Single Exposure Radical Cure and Prophylaxis (abbreviated to SERCaP). Radical in this context refers to the removal of all species of *Plasmodium* in a patient, including the dormant liver stages or hypnozoites and asymptomatic sexual stages or gametocytes. This medicine should wherever possible be given as directly observed therapy (DOT), to ensure compliance, even in challenging field conditions; as such, the SERCaP represents the ideal treatment. It is important to underline that this may not be achievable, and so compromises will undoubtedly have to be made along the way. For this reason this paper provides two definitions, an ideal and a minimally acceptable product. A second class of medicine, a new generation of prophylactics was also suggested, which would be needed in countries which have eliminated malaria, but where a local resurgence of infection could occur.

Historically, MMV played a role in coordinating proposals for TPPs describing both the ideal and minimally acceptable profile for new medicines. The last version concentrated more on the product profiles rather than the candidate profiles, and was produced with the External Scientific Advisory Committee of Medicines for Malaria Venture in 2010. In the present publication, definitions for the attributes of individual molecules (TCPs), and for the final combination product (TPPs) are laid out. The TPPs fall into two groups: medicines which can be used to cure patients, and medicines which can be used to protect populations from infections. The scientific justification and rationale behind these updated recommendations are presented, as well as some of the currently unanswered questions. Strategies for combining the individual candidate molecules to produce the products are discussed, and this is one area where there are potentially many solutions to the same problem. The malaria drug discovery portfolio certainly appears stronger than a decade ago. However, there are now enough data to characterize success rates. This enables a characterization of the unmet needs in terms of how many new molecules will be required in the future.

### General considerations across all TCPs for next-generation malaria medicines

Several characteristics of each candidate molecule are common across all of the different TCPs and can be discussed in general terms.

### Clinical safety and efficacy

Efficacy is initially established in a cell culture model of parasite activity, as close to the human infection as possible. As well as knowing the potency of a new molecule, it is important to also determine its speed of action (the *in vitro* parasite reduction rate), and the stages of the parasite lifecycle where the compound is active, in order to start to estimate how effective it will be in humans [29]. A therapeutic window between the predicted exposure required for a therapeutic effect in the patient, and the no adverse event limit (NOAEL) seen in preclinical safety studies must be established. Since there is sometimes a discussion over whether a physiological change observed is adverse or not, it is useful to also provide the margin compared to the no-effect level (NOEL). The size of this margin needs to be discussed on a case-by-case basis depending on the characteristics of the adverse effect. For example, a three-fold or even lower window may be acceptable if the effects can be monitored and are not considered serious, such as a reversible change in blood chemistry, but even a 100-fold window may not be acceptable in the case of unexplained mortality. This is an area where independent, expert, external review is critical. Molecules must also have a good oral bioavailability, since molecules with bioavailability less than 20% tend to suffer from high variability of exposure, large food effects, and a need for higher doses; these factors have a direct impact on the size of the pill and the cost. The compounds should also have reasonable solubility in gastric fluid, not only because of the impact on bioavailability, but because poorly soluble molecules given at high doses often produce gastrointestinal side effects [30]; this is a problem seen with several current anti-malarial medicines. Since there may be circumstances in which mass drug administration is adopted, particularly in an elimination context, the ideal safety criteria for new medicines is exceedingly high - comparable to that of a vaccine.

### Resistance

Emergence of resistance to treatment is a risk for any infection. The first priority is to determine if the new molecule is active against as wide a selection as possible of the five species of the parasite that infect humans, *Plasmodium falciparum, P. vivax, Plasmodium malariae, P. ovale* and *Plasmodium knowlesi*[31,32], though a pragmatic solution is to focus on *P. falciparum* and, in fewer cases, *P. vivax* due to accessibility to parasites and culture conditions. A second priority is to ensure that there is no cross-resistance against relevant laboratory-adapted strains showing resistance to medicines already in clinical use. Third, it is important to determine the activity of a compound against primary clinical isolates, particularly those from geographical areas known for anti-malarial drug resistance. The final question is to assess the risk of resistance selection *in vitro* to determine how often relevant mutations or amplifications occur, how easily these are selected and what is their fitness cost and transmissibility relative to the wild-type parasites [33]. Such parameters never replace clinical experience, but serve as a guide and risk assessment as to whether new molecules have a high, medium or low propensity to be compromised by resistance.

To minimize the risk of resistance, emerging molecules will be formulated within a fixed-dose combination product. The drugs in a combination should not be cross-resistant with one another, so that resistant parasites to one drug are then killed by the other. No single component of therapy should be exposed to significant numbers of parasites on its own in patients in the field. An ideal for an ‘irresistible’ combination could be to combine two molecules having closely matched human pharmacokinetic cover and potency. However, it may be difficult to find and partner molecules with such matched profiles, but at least the longer-lasting partner should be exposed to as small a number of parasites as possible, once the shorter-lasting drug has disappeared. Ultimately, there is a risk that resistance will emerge to all drugs used against the malaria parasite, but the goal is to have a combination that will withstand resistance pressure for as long as possible during the period of the elimination and eradication agenda, which some have estimated to be a timeframe of at least 50 years.

### Producing an affordable medicine: managing the cost of goods

The manufacturing cost of a new medicine is an important and often-overlooked factor. The goal for a fixed-dose artemisinin combination therapy was an adult dose costing around $1. This has been an important but challenging goal for the research and development community. Current public sector ACT prices are still around $1.50 for the adult treatment, and $0.40 per child. This is an impressive achievement, but even this goal is still some distance from what is affordable to many patients. It would be ideal to have an anti-malarial combination therapy as affordable as a chloroquine treatment was when it was used as monotherapy, costing less than 10 US cents. The challenge of new combination medicines is to keep the costs of each individual component low, as well as minimizing production and packaging costs and hence provide a medicine that is affordable. The foremost factor determining cost is the clinically effective dose in patients: most of the APIs (Active Pharmaceutical Ingredients) in artemisinin combination therapy cost between $100 and $1,000 /kg to produce (at the tonne scale), but some of these are used at total doses as high as 3 g in adults. If new medicines can be found with much lower human effective doses, for example around 30 mg, then the cost of the ingredient would be reduced by 100-fold (all else being equal). As a parenthesis, new generations of molecules with increased *in vivo* potency would also allow new medicines to be tested as slow-release formulations that can be used to achieve longer-term protection. Interestingly, even long half-life oral drugs can benefit from slow release since a well-absorbed drug can gain an additional 24 hours from formulation. For transdermal patches or depot formulations, the active molecules must be hydrophobic, and even for such compounds there is a limit on capacity; currently the maximum dose of any medicine delivered by such technologies is around 10 mg per day [33]. This is a far cry from the current ACT partners, where total drug dosing can be as high as 3.5 g over three days.

**Figure 1:**
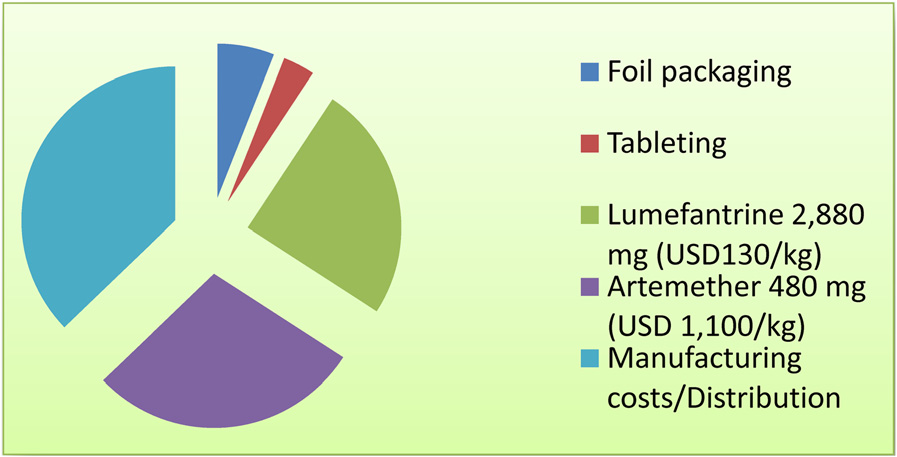
**Example of cost breakdown of artemether lumefantrine ($1.50; R Bryant, *personal communication *for the API costs).**

The ease of synthesis and, ultimately, production is critical: a small number of synthetic steps, each with high yield, from low-cost available starting materials, will also play a role in reducing costs. Lowering the clinical dose generally also reduces variation in exposure and produces fewer side effects, especially the gastrointestinal irritancy caused by high-dose, poorly soluble medicines as discussed above. Packaging and manufacturing costs are often overlooked, and can represent a significant proportion of the overall costs. Compact, simple packaging helps reduce pricing. Figure 1 shows the relative cost structure for a representative anti-malarial medicine [22]. Of note is the fact that absolute packaging costs are similar for small infants as for adults, therefore packaging becomes a larger proportion of the overall cost for the pediatric formulations. These costs would be drastically reduced by cheap single-dose cures, which could be dispensed by healthcare workers. Molecules must demonstrate stability under conditions of high relative humidity and ambient field temperature (37°C and 75% relative humidity), which is quite a different standard to that required for a drug for a Western market. With artemisinin combination therapy there have been examples of endoperoxides reacting with the partner or excipients in the tablet during storage, therefore, requiring bi-layer tablets with inert barriers, an innovative yet more expensive solution.

### Target candidate profiles

**Figure 2:**
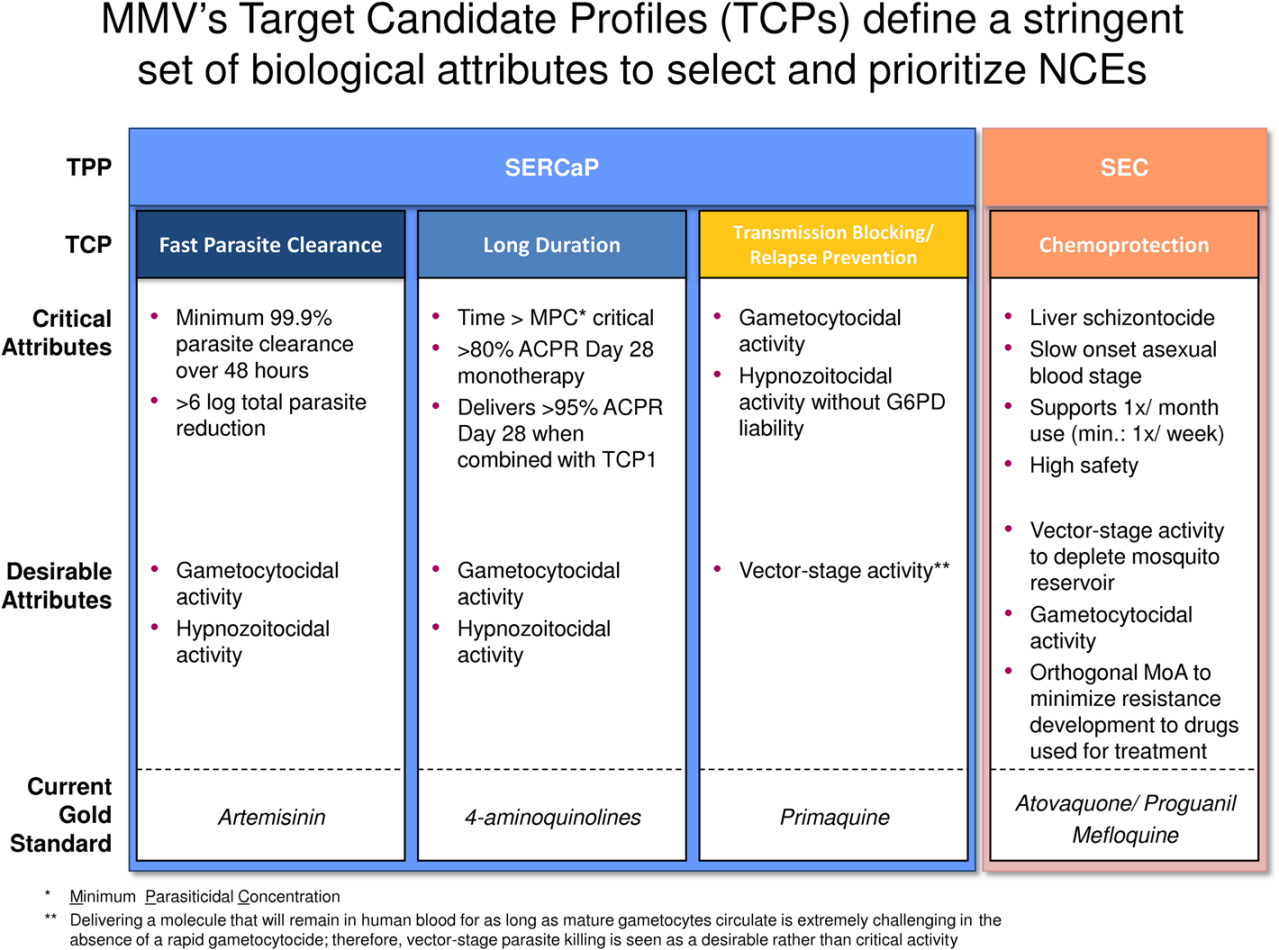
Breakdown of the ideal medicine into different target candidate profiles.

**Figure 3:**
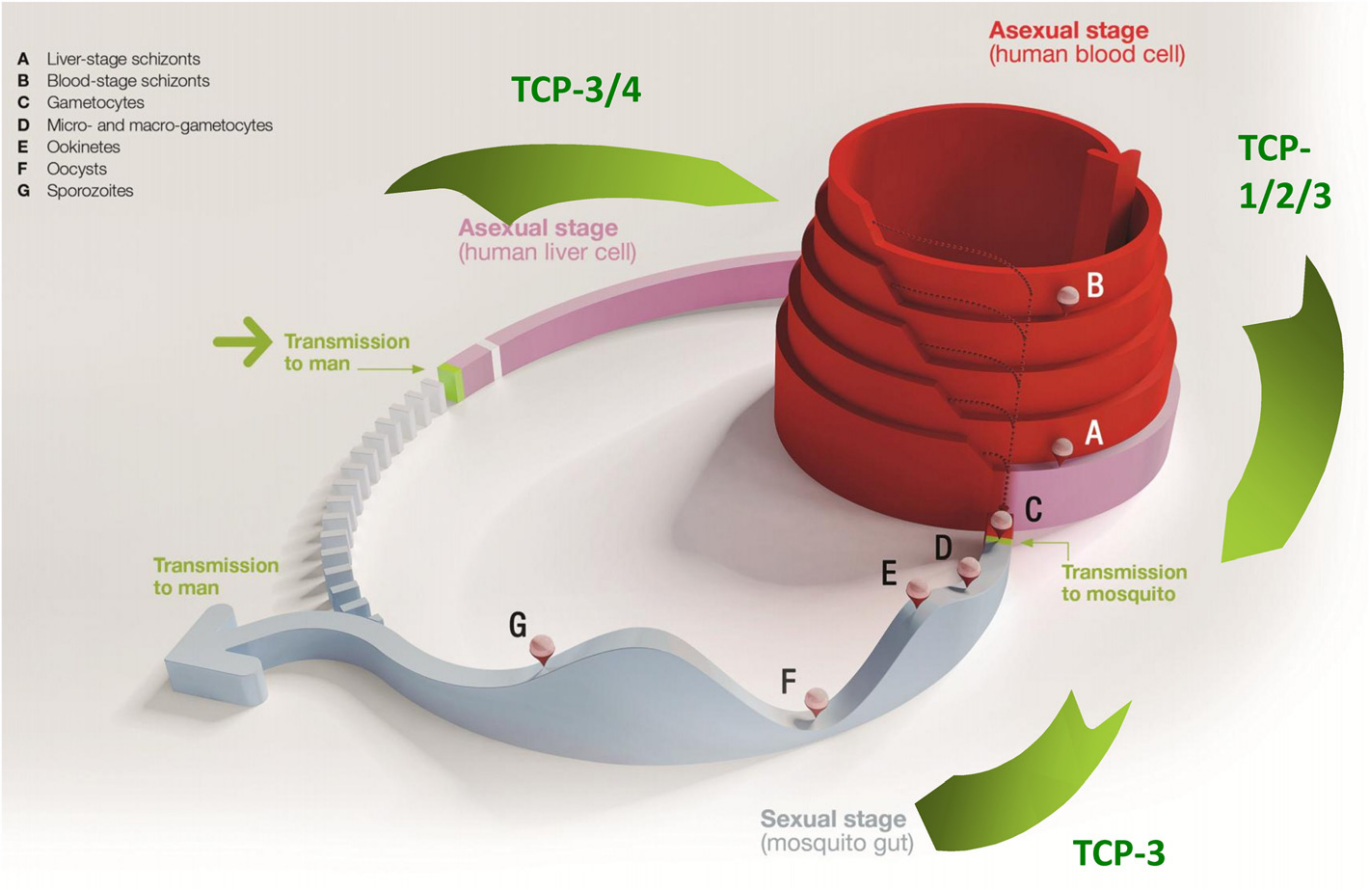
**Diagram of the *Plasmodium *lifecycle and parasite load (z-axis,logarithmic) with stages targeted by the various TCPs.**

The TCPs presented below summarize the four-five types of molecule that are sought to facilitate the elimination and eradication of malaria. Each profile describes a set of attributes for a single compound, for which there should be increasing confidence as a result of the regulatory preclinical studies, and which should be confirmed by the end of the human proof-of-concept (typically phase IIa) trial. Each TCP details a ‘Minimum Essential’ and an ‘Ideal’ profile. The ‘Ideal’ criterion builds on what is described in the ‘Minimum Essential’; therefore, criteria are not repeated unless there is a change. The ‘Ideal’ profile, as stated earlier, requires excellent safety as well as efficacy since administration to asymptomatics, under elimination tactics, is conceivable. It is possible that one molecule may fulfill all the requirements of two different TCPs. This is the case for primaquine which has good clinical activity against *P. vivax* relapse, and can also, with a different dosing regimen, be used to prevent transmission of *Plasmodium*. Figure 2 summarizes the current experience of how these four-five profiles can be combined into a single medicine. Two ideal medicines are described: the ideal treatment or Single Exposure Radical Cure and Prophylaxis (SERCaP), and the ideal chemoprotection or a Single Exposure Chemoprotection (SEC). It is important to underline that these represent ideals, and that during development of combinations then some trade-offs will have to be made. Hence in each case there are definitions of the current view on a minimally acceptable profile. Figure 3 describes how the different TCPs map onto the *Plasmodium* life cycle.

### TCP-1: ‘Fast clearance’, reducing the initial parasite burden

The cornerstone of malaria treatment is the availability of at least one molecule capable of rapidly clearing the parasite load. In order to be effective, a compound addressing TCP-1 (Table 1) would need to remain active for long enough to make a significant impact (>6 log unit reduction) on decreasing the initial parasitaemia. The precise definition of how much activity results in a clinically meaningful reduction in disease, as measured by a decrease in adequate clinical and parasitological response (ACPR) at 28 days after treatment, is still an open question. This is an area where more clinical data on new compounds will help to fill the gap and re-infection as a function of immunity and transmission intensity will need to be factored in. The gold standards for this profile are the artemisinin derivatives, which dramatically lower parasite numbers over three days of treatment by at least four log units, leaving the remaining parasites to be killed by the partner in the combination. An important point here is that this speed of killing needs to be defined in humans: *in vitro* and *in vivo* studies can be used to predict how close any molecule in lead optimization is to the candidate definition, but these are only projections. The MMV experience has been that for success, compounds should typically have *in vitro* activities with an EC_50_ < 10 nM against laboratory-adapted strains and clinical isolates, and a single-digit mg/kg activity ED_90_ in the *P. falciparum* infected human erythrocyte-engrafted SCID mouse model [34]. The rate of clearance of parasites for this TCP is also key; the expectation is that molecules will have a parasite reduction rate (the fold reduction in parasitaemia over one life cycle) at least as fast as 4-aminoquinolines, and ideally faster than artemisinin derivatives. Preclinical models of rates of killing and parasite clearance *in vitro* and *in vivo* can be used to predict this in humans, although these may be underestimated, since they do not allow for immunological or splenic clearance of damaged reticulocytes or erythrocytes [29]. In the ideal case, where the molecule is part of a SERCaP, the molecule would need to produce at least a 10^6^-fold parasite reduction following a single oral encounter of one (or two) doses. For the minimum criteria, a medicine which produces the same effect over two to three days could still represent a clinically relevant alternative to current regimens though would need differentiating qualities to demonstrate advantage and justify investment. This reduction in parasite burden then needs to be confirmed clinically, measured by the proportion of patients who are cured as reflected by the ACPR at day 28. It is still not clear how large a response is required from a single agent to ensure an ACPR of >95% as part of a combination. Lessons from the artemisinins are instructive [35]. Four doses of artemether monotherapy over 48 h leads to a cure rate of just under 50% ACPR at day 28; when combined with lumefantrine over the same dosing period the combined cure rate is >98%. Further studies are currently planned to investigate this clinically with the newer fast clearance compounds, such as OZ439 and NITD609 [6-8], and also to model the ACPR from parasite reduction rates and pharmacokinetics, but this is a work in progress, and no hard and fast rules can be proposed at this stage. The molecule needs ideally to show good activity *in vitro* against the blood stages of all five *Plasmodium* species which infect humans, although activity in *P. falciparum* and *P. vivax* are generally assumed to suffice, particularly since parasites and assays for the others are not readily available. TCP-1 can be summarized in the ideal case as a molecule that should be similar in activity to an artemisinin derivative but with pharmacokinetic properties such that it would allow for less frequent administration and even administration in a single sitting preferably with matched elimination PK and potency with a partner TCP-2 compound.

**Table 1:** TCP-1

**TCP-1 criteria at human proof of concept**	**Minimum essential**	**Ideal**
Dosing regimen; adult dose*	Oral, one-three doses; <1,000 mg	Oral, single dose; <100 mg
Rate of onset of action and clinical parasite reduction ratio from single dose	Immediate and rapid clearance of parasites at least as fast as chloroquine; > 6 log unit total reduction in parasites	Immediate and rapid clearance of parasites at least as fast as artesunate; > 6 log unit total reduction in parasites
Susceptibility to loss of efficacy due to acquired resistance	Low (better than atovaquone); no cross resistance with TCP-2	Very low (similar to chloroquine); no cross resistance with TCP-2. Resistance markers identified
Clinical efficacy from single dose (day 7) including patients from areas known to be drug-resistant to current first line medications	100%	
Clinical efficacy from single dose (ACPR at day 28 or more, per protocol, PCR-corrected)	>50%	>95%
Bioavailability /Food Effect - human data	>30%, <3-fold	>50%, none
Drug- drug interactions	No unmanageable risks	No interactions with other anti-malarial, anti-retroviral or TB medicines
Safety - clinical	Acceptable therapeutic ratio based on human volunteer studies between exposure at human effective dose and NOAEL, dependent on nature of toxicity	Therapeutic ratio >50 fold based on human volunteer studies between exposure at human effective dose and NOAEL; benign safety signal
G6PD (Glucose-6-phosphate dehydrogenase) deficiency status	Measured - No enhanced risk in preclinical data from relevant G6PD deficient animal models	Measured - No enhanced risk in G6PD deficient subjects
Formulation	Acceptable clinical formulation identified	
Cost of active ingredient in final medicine	Similar to current medication: ≤$0.5 for adults, $0.1 for infants under two years	Similar to older medications: <$0.25 for adults, $0.05 for infants under two years
Projected stability of final product under Zone IVb conditions (37^°^C 75% humidity)	≥ 6–24 months	≥1-5 years

*As discussed in the text, should frontline therapies be lost due to reduced efficacy or tolerability then a regimen over 3 days of dosing of novel well tolerated candidates that overcome any resistance will be acceptable.

Finally, it needs to be stressed that in countries or districts where all of the current therapies are incapable of producing an ACPR because of the emergence of resistance, the balance of the objectives changes. The priority in this case would be for molecules which are active against all existing resistant strains, rather than simplification of the treatment regimen.

### TCP-2: long duration partner to complete the clearance of the blood stage parasites

A candidate in this category is a long-acting compound, capable of killing the residual parasites not eliminated by the rapid-clearance TCP-1 medicine (Table 2). Ideally, a compound fulfilling TCP-2 should be able to maintain its plasma concentration above the minimal parasiticidal concentration (MPC) for between two and four weeks (typically needed in high transmission areas with high reinfection rates), thus providing significant post-treatment prophylaxis as measured by non-PCR corrected ACPR at day 28. The MPC is defined as the concentration above which the maximum rate of parasite killing is obtained. This can be measured *in vitro* in a time-dependent viability assay, or *in vivo* by examining the parasite-drug concentration response over time and at different doses. Given the complexities of the biological systems, it is believed that *in vivo* data is likely to be more predictive of the clinical value – which ultimately is measured in a Phase Ib Human Challenge or Phase IIa Clinical study. The total parasite reduction of the current gold standards (4-aminoquinolines and aminoalcohols) is impressive. Compounds such as mefloquine can maintain a blood concentration above the MPC for more than a month, which combined with a parasite reduction rate of 1.5 log units per day, gives an extremely impressive (although theoretical) maximum parasite reduction. The relationship between the duration that MPC is maintained and the post-treatment prophylaxis period is however not clear: mefloquine maintains these concentrations much longer than piperaquine, however piperaquine gives superior post-treatment prophylaxis [36]. Identifying molecules with such a long half-life is a challenge. This is because such drugs normally have high metabolic stability and high affinity for tissue membranes (such as phospholipids); consequently most drugs with exceptional half-lives are lipophilic bases. Such drugs are, therefore, likely to be promiscuous for many human receptors and cause frequent adverse events. In addition they may partake in reversible metabolic clearance steps such as entero-hepatic recirculation, which will contribute to variability and the challenges of development.

**Table 2:** TCP-2

**TCP-2 criteria at phase IIa**	**Minimum essential**	**Ideal**
Dosing regimen; adult dose*	Oral, one-three doses; < 1500 mg	Oral, single dose; < 100 mg
Rate of onset of action and Clinical Parasite Reduction Ratio (PRR)	Dependent on TCP-1 partner. Together with TCP-1 must deliver >95% cure	≥12 log unit reduction in asexual blood stage load. Monotherapy cure
Susceptibility to loss of efficacy due to acquired resistance	Low (better than atovaquone); no cross resistance with TCP-1	Very low (similar to chloroquine); no cross resistance with TCP-1. Resistance markers identified
Clinical efficacy from single dose (ACPR at day 28, per protocol)	>80% PCR-corrected	>95% non PCR-corrected
Bioavailability / food effect - human	> 30%/ < 3-fold food effect	> 50%/ no food effect
Drug-drug interactions	No unmanageable risks	No interactions with other anti-malarial, anti-retroviral or TB medicines
Safety - Clinical	Acceptable therapeutic ratio based on human volunteer studies between exposure at human effective dose and NOAEL, dependent on nature of toxicity)	Therapeutic ratio >50 fold based on human volunteer studies between exposure at human effective dose and NOAEL; benign safety signal
G6PD (Glucose-6-phosphate dehydrogenase) deficiency status	Measured - No enhanced risk in preclinical data from relevant G6PD deficient animal models	Measured - No enhanced risk in G6PD deficient subjects
Formulation	Acceptable clinical formulation identified	
Cost of single treatment	Similar to current medication: < $0.50 for adults, $0.1 for infants under two years	Similar to older medications: < $0.25 for adults, $0.05 for infants under two years
Projected stability of final product under Zone IVb conditions (37°C 75% humidity)	≥ 24 months	≥ 5 years

*As discussed in the text, should frontline therapies be lost due to reduced efficacy or tolerability then a regimen over 3 days of dosing of novel well tolerated candidates that overcome any resistance will be acceptable.

Most compounds used in screening come from diversity collections which have been specifically targeted against diseases in Western markets, for which the goal has often been once daily therapy. Furthermore, molecules with extremely long human half-lives (several weeks) pose additional challenges in development, in terms of the design of toxicological and early clinical studies. A further problem is that the data on fast-killing molecules such as artemisinin, suggest that the logarithmic parasite reduction rates are not additive: a combination of two medicines does not necessarily increase the parasite clearance rate over the fastest compound alone. A simple way of viewing this is that the parasites can only be killed once. The second compound is needed to kill remaining parasites, and during the time that it is present as monotherapy there is a risk of resistance generation whilst the ‘resistance window’ is open. That is when the compound is still above its MPC for wild-type parasite, and hence providing a selective pressure, yet the concentration is below the MPC for any resistant parasite [37].

### TCP-3: targeting *Plasmodium* in the non-dividing parasite stages

As well as possessing erythrocytic-stage killing activity, an ideal combination would need to contain compounds which can prevent the relapse of dormant liver stages (hypnozoites) and the sexual stages of the parasite in the human host or in the mosquito vector. It is possible that a single molecule could be identified with all of these activities. The gold standard for this medicine is primaquine, which is the standard of care for preventing *P. vivax* relapse due to its effects on hypnozoites, as well as its rapid gametocytocidal action [38]. However, primaquine has two characteristics which need to be improved on. First, it needs to be given for 14 days to reliably kill *P. vivax* hypnozoites for radical cure, and although it has been suggested this could be reduced to seven days by increasing the dose [39], there are significant challenges to ensuring compliance with a long course of treatment with a medicine which does not provide any symptomatic relief. Second, it causes significant haemolysis in patients with G6PD deficiency, and shows some gastrointestinal adverse events. An additional concern, resistance to primaquine, has not been clearly observed, although this always remains a background possibility (recently reviewed in [40]). There is some debate about whether the haemolysis is caused by the same reactive intermediate responsible for the effect against the hypnozoite [41,42]. Ideally, a candidate is sought which has parent-derived pharmacodynamics for a single-dose cure and is active against all the non-dividing exo-erythrocytic forms, but without causing the haemolysis; this is described by TCP-3 (Table 3).

**Table 3:** TCP-3

**TCP-3: general considerations**	**Minimum essential**	**Ideal**
Dosing regimen	Oral, once a day for up to 3 days - for use with existing artemisinin-combination therapies (ACTs)	Oral, single dose
Efficacy: TCP3a^a^	Prevents 90% of relapses over a six month period. Human adult dose <1,000 mg	Prevents 90% of relapses over a year. Human adult dose < 100 mg
Efficacy TCP3b	Prevents transmission to mosquito >90% on day 7 post oral dose. Human adult dose <1,000 mg	Prevents transmission to mosquito >90% between 12 h and 7 days post oral dose. Human adult dose <100 mg
Safety	Acceptable therapeutic ratio based on human volunteer studies between exposure at human effective dose and NOAEL, dependent on nature of toxicity)	Therapeutic ratio >50 fold based on human volunteer studies between exposure at human effective dose and NOAEL; benign safety signal
G6PD (Glucose-6-phosphate dehydrogenase) deficiency status	Therapeutic dose identified with change in hemoglobin concentration at day 7 of < 2.5 g/l patients with moderate G6PD activity (60%)	Therapeutic dose shows no significant change in hemoglobin concentration
Drug-drug interactions	No unmanageable risks	No interactions with other anti-malarial, anti-retroviral or TB medicines
Formulation	Acceptable clinical formulation identified	
Cost of single treatment^b^	Similar to current medication: $0.50 for adults, $0.12 for infants for relapse and $0.05 for adults, $0.01 for infants for transmission blocking	Better than current medication: < $0.50 for adults, $0.12 for infants under two years for relapse and < $0.05 for adults, $0.01 for infants for transmission blocking
Projected stability of final product under Zone IVb conditions (37°C 75% humidity)	≥ 24 months	≥ 5 years

Notes:

^a^ Better precision on the clinical efficacy of the gold standard, primaquine in relapse prevention should be available from the phase II comparison with tafenoquine, which will be available in the summer of 2013.

^b^ Estimates of the price elasticity of an anti-relapse therapy are extremely challenging. The price range varies from the cost of treating relapses should they occur and the current treatment costs with primaquine (currently USD $0.04 per 15 mg tablet, so $1.12 for 14 days treatment).

It is of course probable that no new single molecule will be found that can kill both the hypnozoites and prevent transmission, and that these two roles will have to be performed by different molecules in a combination medicine. The anti-hypnozoite attributes needed can be described by a subset of criteria, TCP-3a. Pragmatically, an *in vitro* activity of EC_50_ <100 nM against hypnozoites in a validated assay would be desirable, although ultimately this number could be much higher if the compound has extremely high plasma exposure and is well tolerated in humans. Currently the only system available for testing new medicines uses *Plasmodium cynomolgi* infected primary rhesus hepatocytes, although assays for *P. vivax* infection of human cells are under development ([43]; Sangeeta Bhatia, *personal communication*). The other challenge is the safety margin, in terms of plasma exposure at the EC_90_ and at the no-adverse effect level in pre-clinical species. Treatments should, therefore, be able to prevent relapses in a preclinical animal model or in man without toxicity. *P. cynomolgi-*infected rhesus monkeys have been the model of choice, with a gold standard 8-aminoquinoline as the reference drug [44-46], while new murine models have also been proposed [34,47]. Clinical studies in migrant populations (reviewed in [48]) allow for a relatively simple proof of concept in humans. This ‘out of transmission’ model provides a definitive measure of relapse prevention and it is critical to back-translate and confirm the predictivity and relevance of earlier preclinical models. The attributes needed for clinical transmission-blocking activity are much more difficult to define, but are discussed as a subset of criteria in TCP-3b.

Artemisinin derivatives are capable of rendering stage V gametocytes inactive, but do not prevent malaria transmission clinically on their own due to their short half-lives, and so there is a need for a combination of anti-gametocyte activity combined with long-acting pharmacokinetics or dynamics. There is no generally agreed gold standard of activity required in an anti-gametocyte assay. The standard membrane feeding assay (SMFA) or mouse-mouse transmission models are currently the only intermediate assays that measure formal transmission to the mosquito vector and a mammalian host respectively [49,50]. The additional complication is that primaquine, the gold standard, requires metabolic activation in the liver, and this must therefore be borne in mind when selecting the correct assay. Predicting the efficacy required to achieve clinically significant transmission blockade is not possible with any accuracy at this stage; as an initial guideline it is suggested that molecules should reduce the number of oocysts by >90%, as measured in a clinical study where mosquitoes feed on human blood at various time points post oral dosing. However, this is complicated by the fact that the link between the standard membrane feeding assay and field-based transmission studies still requires further clarification [51]. It is to be expected that better understanding of this will emerge over the next two years as more molecules are characterized in standard membrane feeding assays whose activity can be compared to efficacy in human proof-of-concept studies. The WHO has recently recommended a single dose of 0.25 mg/kg of primaquine as the gold standard for transmission blocking [52]. This single dose is not anticipated to cause significant haemolysis in G6PD-deficient subjects (unlike the 14 day course required for relapse prevention). In the absence of primaquine resistance, this sets a very high barrier for a new molecule to achieve, simply based on transmission blocking alone.

In Table 3, the attributes are described for all TCP-3 molecules, those specific for anti-relapse compounds (TCP-3a) and those for clinically relevant transmission blocking compounds (TCP-3b). The latter group could contain molecules which kill the insect stages of the infection such as oocysts and sporozoites following ingestion of a blood meal. The challenge for such molecules is achieving an effective concentration in the human host for as long as mature infective gametocytes are circulating and so will only be feasible if co-administered with a rapid-acting gametocytocidal agent. Interestingly, transmission blocking can also result from insecticidal activity and ivermectin is currently under evaluation to complement existing tools towards eradication.

Although in an ideal world, where the goal is a SERCaP, there may also be a place for a molecule with both transmission-blocking and anti-relapse activity as part of a three-day course of therapy, together with the current generation of artemisinin combination therapies. Hence for the minimal acceptable profile, a dose given over two or three days could have a role in malaria control and eradication.

### TCP-4: chemoprotection

Ultimately, it would be better to prevent a population from becoming infected rather than treating the patients once they become symptomatic. In many disease areas, vaccines can provide such protection after a single injection providing protection for a large majority of subjects for as much as a decade. No such vaccine has ever been produced for a protozoan parasite, and the history of malaria control has relied successfully on chemoprotection from the earliest days of quinine therapy.

There is a growing consensus on how malaria can be eliminated from affected countries. This strategy consists of active management of cases and their asymptomatic neighbours with first-line therapy in the early stages, followed by more intense programmes to break transmission. These would be followed by measures to contain reintroduction: either case detection by focal screening, or chemoprotection.

As malaria incidence falls, the population in such countries could be expected to move from being semi-immune to being non-immune. Prior to such ‘end-game’ strategies, specific protection of sensitive populations such as pregnant women, infants, or children in zones with seasonal malaria has been shown to have significant impact [12,16]. The challenge, of course, for these preventive medicines, is that their safety profile should be equivalent to vaccination, where serious adverse events in the order of 1:20,000 would be considered problematic, but such a safety profile can only be confirmed several years post launch, and with adequate pharmacovigilance.

Chemoprotection can be achieved by: killing the sporozoite, killing the liver schizonts, or killing the parasites as soon as they emerge into the blood stream from the liver. Chemoprotection might be used to prevent an outbreak from spreading from an introduced index case to neighbouring households, or to protect sensitive populations. The current gold standards for chemoprotection are atovaquone/ proguanil and mefloquine, but both are far from ideal. The frequency with which an anti-malarial needs to be administered to achieve a high level of protection is key when the medicine is used for this purpose. A once per month dosing would provide a significant improvement over the current daily or weekly administrations. A medicine which only needs to be used once per outbreak would have a more significant advantage. Cost will be an important driver: atovaquone/proguanil is a combination daily prophylactic, with an adult cost of $5 per day, although these prices may fall now that the patent protection is expiring. Mefloquine, given one dose per week as mono-protection, is cheaper, costing around $1,000/kg to produce, and so the 500 mg adult weekly treatment costs around $0.50 in raw materials. Cheaper ways to make mefloquine have been developed [53], reducing the active pharmaceutical ingredient (API) cost to around $400/kg, but prices are ultimately linked to volume of demand. Demand may increase if the medicine is shown to have benefit for the prevention of malaria in pregnancy [54]. There is an additional key challenge for TCP-4 (Table 4): in any population the medicines used for suppressive blood stage chemoprophylaxis should be different from that used to treat clinical cases of malaria. Fortunately, TCP-4 does not require the compounds to have a rapid onset of action though since the subject is asymptomatic, and so could include compounds which show a delayed-death phenotype, which have previously been down prioritized for drug development [55].

**Table 4:** TCP-4

**TCP-4 criteria**	**Minimum essential**	**Ideal**
Dosing regimen; adult dose^a^	Oral, once per week; < 1,000 mg	Oral, once per month; < 100 mg
Rate of onset of action	Slow onset of action (>48 h) against asexual blood stages or causal liver stage activity	
Susceptibility to loss of efficacy due to acquired resistance	Very low risk for blood stage	Very low; orthogonal mechanism to treatment use
Clinical protection from infection	>95% protection from primary *Plasmodium* infection	>95% protection from all *Plasmodia* infections (including relapses)
Transmission reduction to the mosquito vector: inhibition of oocysts *via* vector stage targeting at trough levels	No	> 90%
Bioavailability /Food Effect - human data	> 30%, < 3-fold food effect	>50%, no food effect
Drug-Drug Interactions	No unmanageable risks	No interactions with other anti-malarial, anti-retroviral or TB medicines
Safety – Clinical	Acceptable therapeutic ratio based on human volunteer studies between exposure at human effective dose and NOAEL, dependent on nature of toxicity)	Therapeutic ratio >50 fold based on human volunteer studies between exposure at human effective dose and NOAEL; benign safety signal
G6PD (Glucose-6-phosphate dehydrogenase) deficiency status	Measured - No enhanced risk in relevant G6PD deficient animal models	Measured - No enhanced risk in G6PD deficient subjects
Formulation	Acceptable clinical formulation identified	
Cost of single treatment^b^	≥ $0.5 for adults^,^ $0.1 for infants under two years	< $0.25 for adults, $0.05 for infants under two years
Projected stability of final product under Zone IVb conditions (37°C 75% humidity)	≥ 2 years	≥5 yr

^a^ It may be acceptable for a chemoprotectant that is clearly differentiated in other ways versus existing gold standard prophylactics to be dosed more frequently.

An alternative approach to the design of long-acting medicines is the production of a slow-release formulation. In the 1960s this was achieved with cycloguanil pamoate [56,57], where a single depot administration produced long-term protection, but also resulted in the emergence of DHFR (dihydrofolate reductase)-resistant mutant parasites. Such an intramuscular depot would be unacceptable from today’s safety perspective, since it would need surgical removal if there were an adverse event. Developing such technologies for combination therapies would represent an additional challenge.

### Combinations of candidates: a TPP for malaria treatment

**Figure 4:**
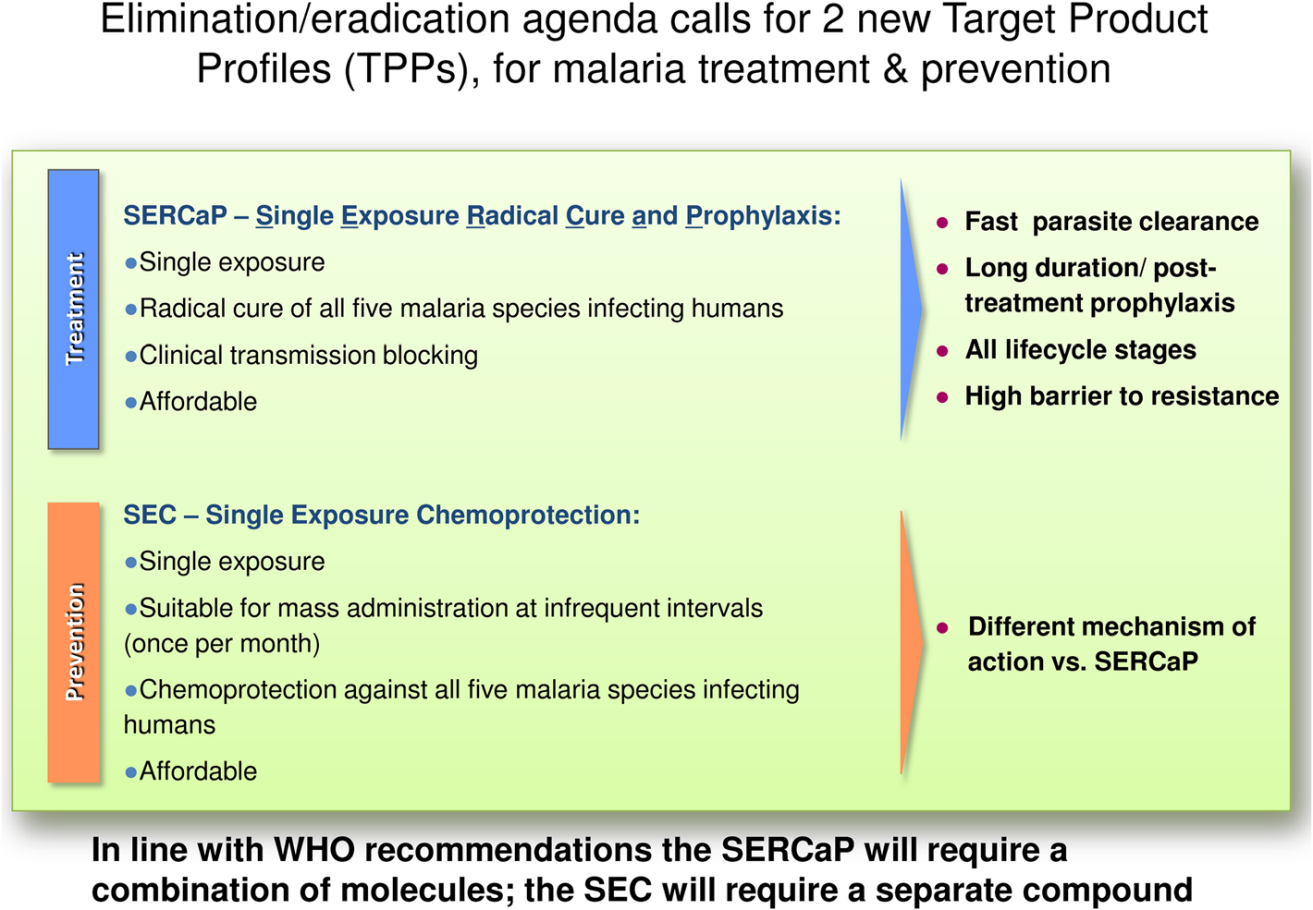
Definition of the TPPs for elimination and eradication.

**Figure 5:**
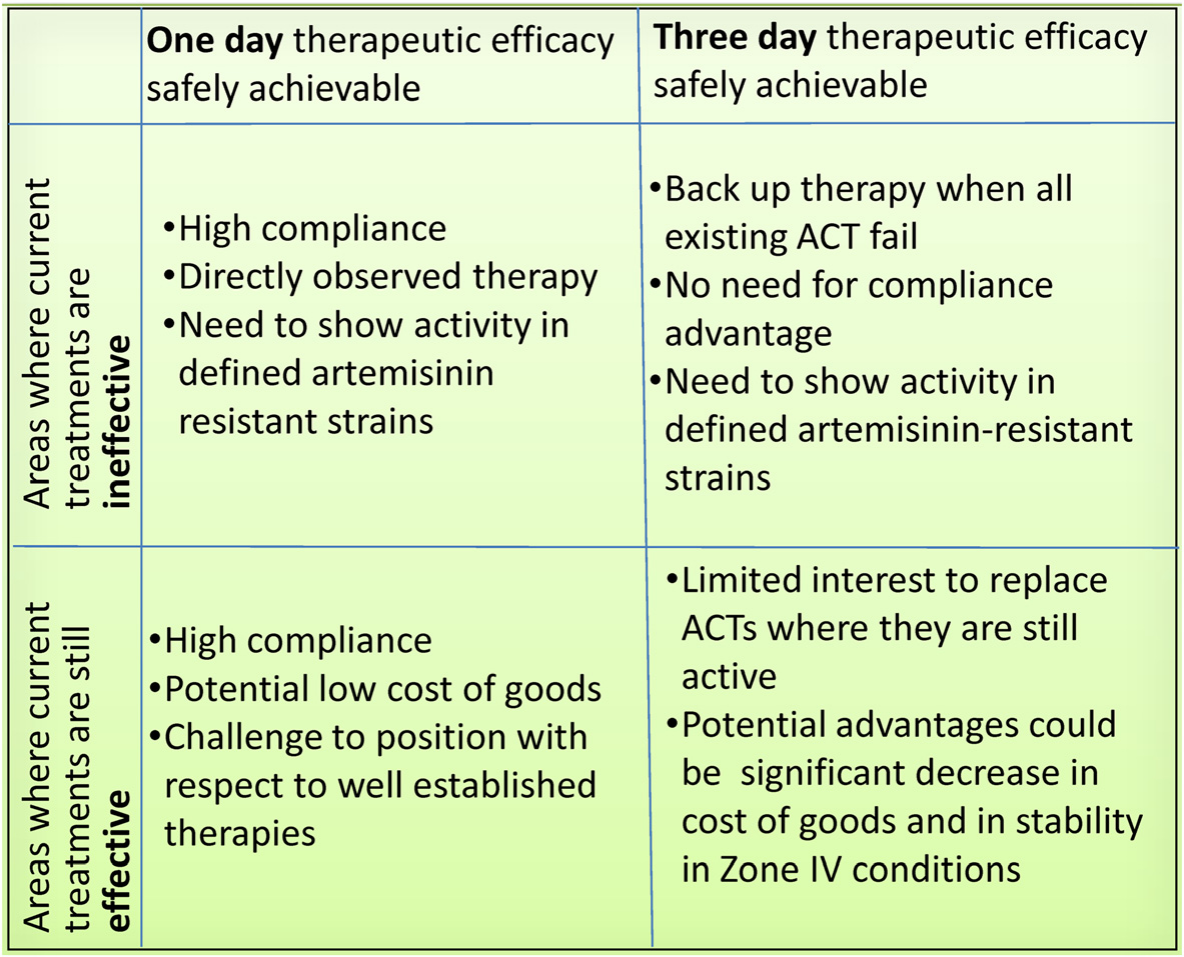
The positioning of new potential therapies, against a background of the competing challenges of the development of 'artemisinin resistance', and the advantages of a single dose cure.

The challenge of combining these candidates to design an ideal medicine against malaria (Table 5) is formidable. There are still many unknown factors, not the least of which is that only a limited number of new classes of molecules have reached clinical evaluation. It is clear that a single molecule can have more than one attribute: a molecule can for example meet the criteria of more than one candidate profile (Figures 4 and 5), but it is essential that combinations of molecules will be needed, not least to combat resistance.

**Table 5:** TPP-1 for the treatment of uncomplicated malaria in children and adults

**Parameter to be demonstrated for the combination in clinical evaluation**	**Minimum essential**	**Ideal SERCaP**
Rate of onset of action	At least one component acts rapidly; patient fever decreased at 24 h	Both components act immediately; patient fever decreased within 24 h
Proportional Reduction in Parasite Load	>12 log unit reduction in asexual blood stage load	
Clinical efficacy (day 7) including patients from areas known to be drug-resistant to current first-line medications	100%	100%
Clinical efficacy (ACPR at day 28 or later, per protocol)	>95% PCR-corrected	> 95% non PCR-corrected
Transmission blocking	No: preclinical models still need to be validated as predictors of clinical outcome	Yes
Relapse prevention: prevents the relapse of *P vivax*, and by inference *P ovale*.	No: preclinical models still need to be validated as predictors of clinical outcome	Yes
		Confirmation in clinical studies capable of distinguishing prevention from delay
Bioavailability/ Food Effect	>30% for each molecule, <3-fold	>50% for each molecule, none
Drug-drug interactions	No unmanageable risk in terms of solid state or pharmacokinetic interactions	No risks in terms of solid state or pharmacokinetic interactions
Dosing regimen	Oral, two-three doses	Oral, once
Safety	Few drug related SAEs in phase III	No drug related SAEs; minimal drug-related AEs
Use in patients with G6PD deficiency	Testing not obligatory due to low risk	No enhanced risk
Pregnancy	Not contra-indicated in second and third trimester	Not contra-indicated
Formulations	Co-formulated tablets or equivalent, with taste masking for pediatrics	Co-formulated tablets for adults. Dispersible or equivalent with taste masking for pediatrics
Cost of treatment course	≤ $1.00 for adults^,^ $0.25 for infants under two years	
Shelf life of formulated product (ICH guidelines for Zones III/IV; combination only)	≥ 2 years	≥ 5 yr
Susceptibility to loss of efficacy due to acquired resistance	Low (better than atovaquone or pyrimethamine monotherapy); no cross resistance	Very low (similar to artemisinin or chloroquine); no cross resistance. Resistance markers identified.

Safety is clearly a paramount concern for any new medicine. The challenge with developing new medicines against malaria is that the current medicines are relatively safe, and serious adverse events rare (less than 1:10,000). This means that any new medicine will be expected to measure up to such a standard, and that in turn requires extensive safety monitoring after launch of a new product. Confirmation that such safety has been achieved will only come with extensive pharmacovigilance, across a wide range of patient ethnicities. In countries planning malaria elimination there has been much discussion of strategies for mass drug administration, or mass screening and treatment (Figure 1). It is important to underline that for mass drug administration the safety profile has to be even more stringent, given the different risk-benefit balance of administering medicines to subjects who may not have the disease. Here, as with vaccines, even 1 in 10,000 adverse events will be problematic. Aiming for a SERCaP places considerable challenges in addition. Giving all the active ingredients as a single dose increases the maximum exposure of each individual molecule, and may reduce the overall clinical safety margin. The benefits of a single dose therapy from a compliance and delivery perspective have to be carefully weighed against the potential risks.

The question of duration of treatment cannot be considered in isolation from the emergence of artemisinin-tolerant strains of the parasite. In countries or districts where artemisinin combination therapies are clinically effective, then clearly the SERCaP brings considerable advantages in terms of directly observed therapy, and potential cost savings, since packaging and distribution will be much simpler (Figure 3). In these countries or districts a new three day course of treatment will offer much less of an advantage. The cost of goods may be lower, but this is set against the extensive clinical safety database for ACT. The rationale for developing a new therapy for this particular segment is much more challenging. However, in the countries and districts where artemisinin combination therapies are no longer effective, because of artemisinin ineffectiveness rather than resistance to the partner, then the scenario is different. Here, a three-day course of treatment with similar safety and efficacy as the current ACT would be acceptable. A single dose cure would still be an advantage, but the risk-benefit calculation would be different. The challenge for drug development is three-fold. Without a molecular biomarker for ‘artemisinin resistance’, it is difficult to assess currently how many people fall into this high-risk group. Second, in any case, there are no models showing how the population which cannot be effectively treated with ACT will develop over the next decade. Third, currently there are not sufficient numbers of patients with reduced parasite clearance rates to enable clinical studies of new therapies, and in any case the public health priority is to eliminate the parasite in these regions.

After considerations of safety and efficacy, the principal concern for a SERCaP will be to avoid the development of resistance. If the SERCaP is to help in driving malaria eradication it would be best if it did not have to be regularly cupgraded’, as happens with many vaccines against common bacterial or viral pathogens, and some drugs too. To avoid resistance, the key is to make sure that no one molecule is exposed to a large number of parasites on its own. The way this is achieved with an artemisinin combination therapy is that the artemisinin analogue reduces the parasite numbers by at least 4 log units over a three-day course, though this still leaves a maximum of 10^8^ parasites for the partner to face alone. The closest current gold standard combination against which to judge a SERCaP would therefore be an ACT plus primaquine (to prevent transmission). This is a combination of TCP-1, -2 and -3b. However, an ACT plus primaquine fails to meet the TPP because single dose primaquine does not prevent relapse. Other combinations are of course possible. The problem of leaving a partner to face the parasite alone is mitigated by having TCP1s with higher rates of parasite clearance than the artemisinins (clearly a major challenge) or extended durations of exposure (to ensure a greater overall reduction in parasite burden). The ultimate mitigation, however, is a strategy of matched pharmacokinetics and potency (for example a combination of two TCP-1 molecules plus a TCP-3, all with similar pharmacokinetic-potency characteristics). Clinical data suggests that logarithmic additivity in parasite killing activity should not be assumed with anti-malarial combination treatments: for example, the parasite reduction over time with artesunate-mefloquine is no faster than artesunate [58]. These observations are also reflected in *in vitro* measurements of the parasite reduction rate with combinations (L. Sanz, *unpublished data)*. Thus, for compounds with matched pharmacokinetics where no logarithmic additivity is seen, both molecules are likely to need to achieve a PCR-corrected ACPR of greater than 95% as single agents. This additional stringency may make it difficult to identify suitable candidates. Should additivity be observed, as a result of complementary stage specific action then the individual ACPR will be less. In addition, a combination of two short-acting molecules will provide poor post-treatment prophylaxis and hence not deliver a formal SERCaP; operationally this could be a major disadvantage in high-transmission areas. Another interesting question is whether the gametocyte-killing activity needs to be in the TCP-3 molecule; a TCP-1 molecule with additional anti-gametocyte properties would allow a TCP-1/3b, TCP-2, TCP-3a combination, for example. Although several of the new fast-killing TCP-1 candidates have highly potent activity against stage V gametocytes, it is not clear yet whether this is sufficient to block transmission in a clinically meaningful way. Artemether is an excellent killer of gametocytes in culture, but artemether-lumefantrine does not successfully block transmission on its own, presumably due to the poor pharmacokinetics of the artemisinins [51,59].

### A TPP for a new medicine for chemoprotection

In any disease eradication agenda, preventing the population from becoming infected is a key activity. In malaria this has been primarily achieved to date with bed nets. Vaccination is another strategy, but apicomplexan parasites have developed sophisticated immuno-evasive strategies. Chemoprotective medicines offer an additional approach to disease control (Table 6). These medicines could be used to protect vulnerable populations, and also in the situation where there was an outbreak of malaria in an area previously shown to be malaria-free. This medicine would contain a combination of two anti-malarial APIs based on TCP-4 since its widespread use would raise significant concerns about resistance emerging if used alone. Since prophylaxis can come from causal or suppressive activity it is ideal if the combinations partners target the same parasite stage. It is preferable for the medicine to be given infrequently. Current chemoprotection regimens in children are given monthly throughout the season. The technical challenge of developing a medicine which can protect for several weeks is enormous, and will require extensive safety studies. Within the chemoprotection concept are also the medicines for intermittent presumptive treatment for pregnancy (IPTp) and its equivalent in infants (termed IPTi) and children, (termed either IPTc) or seasonal malaria chemoprotection. Over the next decade, these therapies are most likely to involve new combinations of existing registered medicines, but in the longer term new classes of medicines will be needed. Cost is an important driver here: as the incidence of malaria falls to a level where elimination is feasible or achieved then the cost-benefit ratio of chemoprotection increases.

**Table 6:** TPP-2 for a new medicine for chemoprotection

**Parameter to be demonstrated for the combination in clinical evaluation**	**Minimum essential**	**Ideal SEC**
Dosing regimen	Oral, once per week	Oral, once per month
Rate of onset of action	For asexual blood stage action – slow onset (48 h) - before rapid killing	
Clinical efficacy	Prevents primary infection of *Plasmodium* >95%	Prevents *Plasmodium* infection including relapse >95%
Transmission blocking	No	Yes
Bioavailability/ Food Effect	>30% for each molecule, <3-fold	>50% for each molecule, none
Drug-drug interactions	No unmanageable risk in terms of solid state or pharmacokinetic interactions	No risks in terms of solid state or pharmacokinetic interactions
Safety	Few drug related SAEs in phase III	No drug related SAEs; minimal drug-related AEs
Use in patients with G6PD deficiency	Testing not obligatory due to low risk	No enhanced risk
Pregnancy	Not contra-indicated in second and third trimester	Not contra-indicated
Formulations	Co-formulated tablets or equivalent, with taste masking for pediatrics	Co-formulated tablets for adults. Dispersible or equivalent with taste masking for pediatrics
Cost of treatment course	≤ $1.00 for adults^,^ $0.25 for infants under two years	
Shelf life of formulated product (ICH guidelines for Zones III/IV; combination only)	≥ 2 years	≥ 5 yr
Susceptibility to loss of efficacy due to acquired resistance	Very low; no cross resistance with partner	Very low; no cross resistance and orthogonal mechanism from those used in treatment

### Other TPPs: severe malaria

The standard of care for severe falciparum malaria including cerebral malaria is now shifting from quinine to parenteral artesunate, based on recent clinical results obtained in South-East Asia and in sub-Saharan Africa [60,61]. The prevalence of severe malaria will fall as the total malaria numbers drop, but the proportion of cases that are severe may increase as the population loses some of its immunity, and severe malaria will remain a challenge until the very end of the eradication agenda. As the frequency of malaria cases falls, the risk of late or even incorrect diagnosis increases (as is seen in European travellers who return home), increasing the risk of severe disease that is not or inappropriately treated. A number of considerations apply to a new treatment for severe malaria. First, in the absence of a failure of artemisinin treatment due to acquired drug resistance it is unlikely that a new therapy could demonstrate clinical superiority over artesunate, since this would require extremely large numbers of severely ill patients (probably more than 10,000). Second, severe malaria patients are by definition fragile. Before a new medicine is tested in this group of patients it must already be known to be safe and efficacious against un-complicated malaria, otherwise there may be undue risks to the patient. If new medicines for treatment of severe malaria become necessary because of the widespread failure of artemisinins it is likely that either *i.v.* quinine will be reinstigated or the subset of TCP-1 molecules, already shown to be effective in uncomplicated malaria and for which an intravenous formulation is feasible, will be investigated. This is a special case: monotherapy would be considered adequate, since all patients would be treated afterwards with an oral combination therapy. Not all TCP-1 molecules will fall into this class, since some of them may not be sufficiently soluble for parenteral use. Third, while there is clearly a need for adjunct therapy to minimize the sequelae of severe malaria, these molecules will already have been shown to have clinical efficacy and high tolerability in studies before they are tested in children with severe malaria. The search for such medicines will largely come from investigators working in other therapeutic areas.

### How many candidate molecules are needed to produce the next generation of medicines?

The increased investment in research and development of new medicines over the last decade has increased both the strength and the diversity of the global portfolio of anti-malarial medicines. The portfolio contains many new chemotypes currently being tested in regulatory non-clinical studies for the first time, all of which have been discovered in the last six years [17]. In the last year, three new medicines from the global portfolio progressed into formal preclinical development, and the evidence is that this trend is sustainable provided investment is maintained. The results from high-throughput screening against living parasites, and the willingness of the community to allow their existing large chemical collections to be screened in assays developed by others, gives confidence that this trend in the discovery of new molecules can continue, provided that the resources are available. A key question is how many new molecules are needed to properly meet future clinical needs as discussed previously, given the attrition rates in clinical development. Benchmark data for success rates in drug discovery and development are often difficult to interpret, since they are always based on past successes. Across the pharmaceutical industry data are collected by the Centre for Medicines Research (CMR), but these cover a wide spectrum of infectious disease, and may miss malaria-specific details. MMV has collected data from the malaria drug discovery and development projects that it has been involved with over the last ten years, which are fairly similar to those from the CMR, but reflect a much smaller sample size and also the ‘Me too’ nature of the late-stage development portfolio. These data are summarized in Table 7, which shows a 12% success rate for preclinical candidates becoming part of a launched medicine for MMV and a 4.4% success rate for CMR. The lower number for CMR reflects the recent difficulty in developing new classes of antibiotics [62].

**Table 7:** Success rates and costs for development of an anti-malarial medicine (2007–12), compared with benchmark data from the Centres for Medicines Research (2008–11)

**Stage**	**Cumulative success rates (CMR)**	**Cumulative success rates (MMV)**	**Cost per stage (MMV)**^**b **^**/ millions USD**
Preclinical	4.4%	12%	1.8
Phase I	8%	23%	1.5
Phase IIa	15%	34%	5.4
Drug interactions/ Phase IIb	51%^a^	60%	8.7
Phase III	68%	80%	31.0^c^
Submission	96%	100%	2.0

Notes:

^a^A stage success rate of 75% for combining two medicines has been added in to reflect the potential for unfavourable drug-drug interactions that prevent further development of a combination. The same correction has been used for CMR and MMV success rates, although our experience of these studies is currently not sufficiently large to enable this to be accurately estimated.

^b^The cost per phase is based on MMV project costs, and does not allow for in-kind contributions from our pharmaceutical partners, or for the internal MMV staff costs.

^c^The estimate for phase III costs is taken from the pyronaridine-artesunate project, where all the clinical costs were borne by MMV, and four pivotal studies were carried out. This does not include internal project management costs.

In most disease areas success rates are tending to fall over the long term, reflecting difficulties in target validation at one end of the drug discovery process, and increasing stringency from the regulatory authorities at the other end; the yearly rate of new drug approvals (all areas) has remained mostly flat over the past 60 years, in spite of tremendous increases in expenditure on R&D [63]. For neglected diseases, the use of whole parasite screening avoids wasting efforts on non-valid targets, and the close collaborative interactions with the regulatory authorities mean that there is a certain confidence that our success rates, in established areas, will not fall dramatically. If the success rate remains unchanged one can predict the number of drug candidates that need to be developed in order to produce a new drug. The overall probability P for ending up with at least one launched product starting with n candidates with individual success probability s is described by the equation (1- P) = (1- s)^n^, or n = log(1 – P)/log(1 – s), as derived from the Negative Binomial Distribution. Using MMV’s empirical value for s (0.12, see Table 7), and aiming for a 90% overall probability of success (P) this would give us a requirement for 18 candidate molecules to result in one launched product. There is some level of confidence for these probabilities as they are based on the experience of finding molecules which have fast-killing activity of blood stages. However, for the transmission-blocking and anti-relapse compounds it is difficult to be so confident, since there is much less validation that the associated *in vitro* assays can be used to predict clinical reality, and in addition the parasites are generally non-dividing at this stage and therefore have a narrower range of potential molecular targets. The long-term development of a triple-combination medicine containing three new molecules would require as many as 30–40 new candidate molecules, in a world where as a global drug discovery community we are discovering only two or three new candidates per year. Even with the current strong portfolio there is still a need for at least another decade of drug discovery, and another one of development beyond that.

## Discussion

The call for an Agenda for Malaria Eradication has set new challenges for all those engaged in the drug discovery process. New medicines are needed to back up the current gold standard ACT therapies, so as to provide immediate alternative control strategies should the reduced speed of action of artemisinin spread. In addition, new medicines are needed to prevent transmission and relapse, the causes of new disease episodes. Finally, all medicines have to be as safe and convenient and cost-effective as possible, which represents an additional challenge. These increased demands are set against a background of two additional difficulties. First, the overall resource of drug discovery in neglected diseases is still relatively small, and in any case the overall productivity of drug discovery (for all indications) is decreasing. Second, the availability of human, *Anopheles* and *Plasmodium* genomic information has not had an immediate impact; progress in biological understanding paradoxically has led to overshooting rational drug discovery efforts with excessive confidence in the power of reductionism. On the positive side, success rates for finding new chemical series have increased with improvements in high-throughput screening using live parasites and the use of large, wide-diversity compound collections, such that now there is an abundance of new chemical series to work on [17]. These hits are now in turn yielding new targets, often previously understudied by the community, which will help set an agenda for more mechanism-based approaches with higher confidence in target validation in the future.

The availability of new chemical series to work on has underscored the need for clarity about the type of molecules which are needed to achieve the malaria eradication agenda. These goals are essential to guide the medicinal chemistry process needed to identify appropriate candidates. Building on existing therapies and future clinical needs, TCPs have been described for a rapid-onset molecule, a long-acting molecule, one that prevents relapse and stops transmission, and one that will act as a chemoprotectant. An analysis of the current success rates of drug development shows that for a 90% chance of registering a molecule as many as 20 different preclinical candidates may be required, of which some already exist. The challenge is that much of the existing portfolio is focused on the TCP-1, the rapid-clearance molecule. This has led to a relative dearth of new molecules with long half-lives, those which can kill the non-dividing forms of the parasite, and those active against sexual stages of the parasite. One of the priorities for anti-malarial drug discovery is to ensure that there is a standardized measurement of the activity of clinical candidates across the whole life cycle of the parasite, termed the Malaria Life cycle Fingerprint [64].

The way in which candidate molecules are combined to fulfill the final target product profiles shows that there is a certain amount of flexibility depending on the actual attributes of the molecules themselves. Theoretically, several different ways of configuring a new combination medicine can be envisioned. These include the combination of the first three target candidate profiles (TCP-1, -2 and −3), preferably with matching half-lives and even combinations which allow for one molecule having more than one attribute (TCP-1/3b, TCP-2, TCP-3a). However, in discussions of potential combinations the reality is often more simple than the theory. Discussions about potential partnering strategies for new molecules in phase IIa, such as the endoperoxide OZ439 [6,7] or the spiroindolone NITD609 [8], highlight that these molecules can only be combined with molecules which have already been shown to be active in phase IIa. This largely limits the choice of TCP-2 candidates to the known 4-aminoquinolines or amino-alcohols, or to molecules of antibacterial origin. Each of the 4-aminoquinolines or amino-alcohols has strengths and weaknesses in terms of half-life, cost, pre-existing resistance and dosing.

However the process of reviewing potential partners underlines the need for other new classes of TCP-2 candidates for the future. These will not be easy to find, since the chemical diversity currently available for screening is focused more around medicines that can be given once per day. However, the availability of over 20,000 new hits which kill the parasite may allow the sub-selection or hit optimization of molecules with a long half-life; these types of prioritization will become increasingly important over the next few years. The other alternative is to combine two fast acting compounds, for example the two new agents OZ439 and NITD609. This has the advantage of using molecules which have never been exposed to malaria as single agents, which has a certain appeal. The plasma exposures of both molecules remain above the minimum parasiticidal concentration for around a week, and so it would not be expected that such a combination would provide the same post-treatment prophylaxis as the current ACT, although this is less of an issue in low-transmission settings since reinfection rates are lower.

The choice of TCP-3 molecules (preventing relapse and transmission) is even more stark. Currently the only option is the 8-aminoquinoline primaquine, which requires 14 days of therapy. An analogue, tafenoquine, is in clinical trials to determine whether it can be efficacious and safer than primaquine as a single dose. New families of active molecules are starting to be prioritized, but so far none has reached clinical development. It is important to underline that even with substantial investment in this area there are unlikely to be new molecules with clinically proven activity within the next five years.

Putting all these molecules together to achieve a single-exposure radical cure and prophylaxis is clearly the ideal situation, but may be difficult to attain. To ensure adequate coverage from a single exposure, the dose of each component will have to be high, and this inevitably reduces the safety margin of the product. It is important to underline that whereas an ambitious objective is laudable, then less dramatic improvements in the regimen (such as two doses in a day, or two days of dosing) still represent a step in the right direction and should not be discarded. In the regions where ACT is failing to provide adequate treatment, then a three-day regimen would be clinically advantageous.

The identification of TCP-4 as the cornerstone of the chemoprotection agenda is also a critical issue. Once again, there are few candidate molecules in the pipeline, and this is an issue that has to be redressed. However, there are also grounds for hope here. The fact that rapid onset of action and fast killing are not required means that there are already several scaffolds and target types which could be studied for their relevance to TCP-4, including previously discarded compounds with a delayed-death mode of action. New medicines for chemoprotection must be tested for their effects amongst people in malaria-endemic areas, and designed for use by people in those areas, rather than tourists or travellers.

## Conclusions

The Agenda for Malaria Eradication has set ambitious goals for the treatment and chemoprevention of malaria, which cannot be reached with the currently available medicines. The combination of the complexity of drug discovery and development, plus a timeline of over a decade from discovery to launch in the first country, means that clarity at the start of the process is critical for success. Acceptable and ideal TPP for a treatment and chemoprotection agent have been defined. These have then been broken down into constituent parts - defined by the respective TCPs. As with any retrosynthetic process, there are a number of different ways the product can be broken down, and the final one chosen will depend on the ease of identifying suitable molecules for each TCP. The definition of target candidate profiles has highlighted the extreme shortage of molecules for three of the four profiles. Whilst there is some ground for optimism that this gap will be closed over the next decade, it will require a focused effort by the whole malaria drug discovery community as well as a sustained source of funding. In addition, well-validated, robust, functional assays for hypnozoites and transmission blocking activity with proven clinical correlations are required. Keeping a continued focus of the community on such challenging end-goals, through the TPPs, helps to ensure that the final products are in line with the patient and public health needs of the future. With such a focus, the community should be able to partner to deliver new medicines with clinical improvements over the current gold standards, and lead the way in the eradication of malaria.

## Abbreviations

ACPR: Adequate clinical and parasitological response; ACT: Artemisinin-combination therapy; API: Active Pharmaceutical Ingredient; ATQ: Atovaquone; CMR: Centre for Medicines Research; DHFR: Dihydrofolate reductase; DOT: Directly observed therapy; EC/D90: Concentration/dose that results in 90% suppression of parasitaemia; G6PD: Glucose-6-phosphate dehydrogenase; HIV: Human immune deficiency virus; ICH: International conference on harmonization; IPTc: Intermittent preventive treatment in children; IPTi: Intermittent preventive treatment in infants; IPTp: Intermittent preventive treatment in pregnancy; IRS: Indoor residual spraying; malERA: Malaria eradication agenda; MMV: Medicines for malaria venture; MPC: Minimum parasiticidal concentration; NOAEL: No adverse effect level; PRR: Parasite reduction ratio; RH: Relative humidity; SAEs: Severe adverse effects; SEC: Single exposure chemoprotection; SERCaP: Single exposure radical cure and prophylaxis; TB: Tuberculosis.

## Competing interests

The authors declare that they have no competing interests, beyond the fact that MMV is involved in supporting the development of some of these medicines.

## Authors’ contributions

JNB, JJM and TNCW composed the TCP Tables - CO analyzed malaria drug discovery attrition, TNCW wrote the initial manuscript draft, and all authors contributed with further edits, comments and discussion. All authors read and approved the final manuscript.
